# Elastic-Net Copula Granger Causality for Inference of Biological Networks

**DOI:** 10.1371/journal.pone.0165612

**Published:** 2016-10-28

**Authors:** Mohammad Shaheryar Furqan, Mohammad Yakoob Siyal

**Affiliations:** 1 School of Electrical and Electronic Engineering, Nanyang Technological University, Singapore, Singapore; 2 INFINITUS, Infocomm Centre of Excellence, Nanyang Technological University, Singapore, Singapore; Instituto Nacional de Medicina Genomica, MEXICO

## Abstract

**Aim:**

In bioinformatics, the inference of biological networks is one of the most active research areas. It involves decoding various complex biological networks that are responsible for performing diverse functions in human body. Among these networks analysis, most of the research focus is towards understanding effective brain connectivity and gene networks in order to cure and prevent related diseases like Alzheimer and cancer respectively. However, with recent advances in data procurement technology, such as DNA microarray analysis and fMRI that can simultaneously process a large amount of data, it yields high-dimensional data sets. These high dimensional dataset analyses possess challenges for the analyst.

**Background:**

Traditional methods of Granger causality inference use ordinary least-squares methods for structure estimation, which confront dimensionality issues when applied to high-dimensional data. Apart from dimensionality issues, most existing methods were designed to capture only the linear inferences from time series data.

**Method and Conclusion:**

In this paper, we address the issues involved in assessing Granger causality for both linear and nonlinear high-dimensional data by proposing an elegant form of the existing LASSO-based method that we call “Elastic-Net Copula Granger causality”. This method provides a more stable way to infer biological networks which has been verified using rigorous experimentation. We have compared the proposed method with the existing method and demonstrated that this new strategy outperforms the existing method on all measures: precision, false detection rate, recall, and F1 score. We have also applied both methods to real HeLa cell data and StarPlus fMRI datasets and presented a comparison of the effectiveness of both methods.

## 1 Introduction

In the modern age of bioinformatics, scientists are endeavoring to find ways to cure diseases at their source, making the recuperation process faster and more efficient. For this reason, researchers from diverse areas are striving to comprehend and replicate complex networks involved in the operation of various functions in human body. Among those networks, most of the research is focused on mapping of effective brain connectivity in the brain for specific task and gene networks in the translation of different biological reactions.

### 1.1 Brain connectivity

The brain connectivity analysis is crucial for exploring the network topology and understanding of the inter- and intra-communications involved during execution of any task as brain function does not involve isolated regions but rather requires a network of various regions to perform any task [1]. This motivated the researchers to develop the means to extract and replicate that network information.

The review by Firston [2] and others [3] divided the brain connectivity studies into three distinctive branches namely structural, functional, and effective connectivity. The Structural connectivity analysis involves the study of the anatomical links of fiber tracks that associate the neuron pools across different brain regions. Functional connectivity maps the region of the brains that are spatially distributed, but functionally connected. These functional maps are generated using statistical concepts that capture the deviation of statistical independence. However, the effective Connectivity represents an amalgamation of structural and functional connectivity showing the directional effects within a network pool.

### 1.2 Gene Networks

Gene is the basic physical and functional unit of heredity that communicates and interacts with each other to make proteins that help in performing various biological functions. Thus motivating researchers to obtain a better understanding of protein’s functional interactions which provide exceptionally valuable information for discovering susceptibilities of a disease to its treatment.

In recent times, a wide range of methods for network analysis have been developed to detect the brain connectivity and gene networks that use time series data extracted from fMRI and DNA microarray. These time series data can be analyzed by utilizing a number of techniques from various fields such as econometrics. Among several techniques, Granger causality is ubiquitously used in biological network analysis (gene network analysis [4–7] and mapping of effective brain connectivity [8–12]) because of its simplicity in terms of its implementation and interpretation [13, 14]. However, its use faces limitations when dealing with high dimensional data.

The standard implementation of Granger causality as proposed in [15] was originally developed with the aim of analyzing direct or linear causality by using ordinary least-squares (OLS) methods for causality estimation. However, the use of OLS implementation limits its use for the high-dimensional biological dataset. Therefore, in this paper, we are proposing a new method based on the elastic net and copula approaches for finding the Granger causality for high-dimensional data. The proposed method will not only be able to detect direct causality but will also analyze indirect causality as well.

This paper first reviews the concept of Granger causality, its existing implementations, and their limitations in Section 2. In Section 3, we present our proposed method for addressing these limitations. Section 4 covers the experimental details and results used to evaluate the performance of the proposed method. Finally, the discussion and conclusion are presented in Section 5.

## 2 Granger Causality

Granger causality analysis was first introduced in econometrics [15] for studying causal relationships using different financial time series data. The classical literature, such as New Introduction to Multiple Time Series Analysis by Lütkepohl [16], showed that Granger causality can be applied using many modeling techniques such as vector autoregressive (VAR) models, infinite order VAR, impulsive response functions and more. However, the literature in bioinformatics indicates that the VAR modeling technique is commonly used because of its simplicity and ease of interpretation [17].

To understand the concept of Granger causality, let us consider two-time series, *X*, and *Y*. If previous values of both *X* and *Y* can be used to predict the current value of *X*, i.e., *X*[*n*], then we can conclude that *Y* Granger-causes *X*. Thus, including both variables increases the prediction’s effectiveness over that of using past values of *X* alone [15, 16].

**Fig 1:**
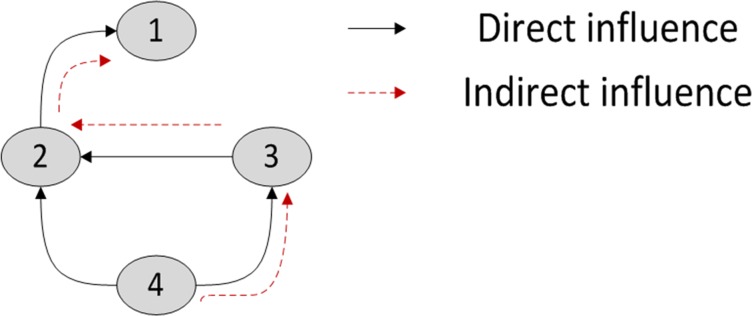
Direct and indirect influence.

Granger causality can be used to explain direct or indirect influences. To understand the concept of direct (linear) influence and indirect (nonlinear) influence, consider a simple four-variable scenario as shown in Fig 1. Direct influence is represented by direct links between nodes, as exemplified by the edge between Node 2 and Node 1, whereas indirect influence can be traced using the edge that is mediated by one or more nodes. In Fig 1, an indirect influence can be observed by tracing the edge from Node 4 to Node 1, mediated by Node 3 and Node 2. If either Node 2 or Node 3 is blocked, then Node 4 will have no effect on Node 1.

Inference of biological networks using high-dimensional data extracted via various techniques confronts two critical challenges in finding the temporal causal relationships: 1) not all significant confounders in the data sets are known and 2) a large number of high-dimensional time series need to be analyzed.

The first challenge centers on the fact that most data sets do not measure all confounders, which makes surreptitious effects due to unobserved confounders unavoidable. In this circumstance, prior knowledge of unobserved confounders can be utilized to overshadow their effect.

The second challenge requires us to develop adaptive algorithms that can reveal temporal dependencies by utilizing a substantial number of time series that have few observation values.

In the past, several methods were used to handle such high-dimensional data, such as pairwise analysis [18], kernel-based algorithms [11, 19], and other regularization-based methods [20, 21]. Another viable alternative, proposed by Nelsen [22], uses the copula to discover dependencies between random variables. The use of probability theory in the form of copulas counters the spurious effect of confounders by utilizing marginal probabilities to incorporate prior information about them.

Recently, Liu, Lafferty, and Wasserman [23] introduced the Gaussian copula with nonparametric marginals, which can be used to estimate high-dimensional undirected graphs for inferring influences between high-dimensional time series. This idea was taken forward by Bahadori and Liu [24], who proposed a new method for finding Granger causality, called the Gaussian non-paranormal (GNPN) model.

They defined the GNPN model by considering a time series *X* = (*X*_1_, …, *X*_*n*_) having a GNPN distribution of *GNPN*(*X*,*B*,*F*) as long as there are functions ${\left\{F_{j}\right\}}_{j=1}^{n}$ such that *F*_*j*_(*X*_*j*_) for *j* = 1, …, *n* are jointly Gaussian and can be factored according to the VAR model through coefficient *B* = {*β*_*i*,*j*_}. To be more precise, variables $Z_{i}\hat{=}F_{j}(X_{j})$ can be factored as shown below:

$$p_{z}(z)=N(z(1,\ldots ,L))\times \prod_{j=1}^{n}\prod_{t=L+1}^{T}p_{N}(z_{j}(t)|\sum_{i,j}^{T}\beta_{i,j}^{T}z_{i}^{t,Lagged},\sigma_{j})$$

where *p*_*N*_(*z|μ*,*σ*) is the Gaussian density function having variance *σ*^2^ and mean *μ*, *L* is the maximal time delay, *z*_*i*_^*t*,Lagged^ = [*z*_*i*_(*t* − *L*), …, *z*_*i*_(*t* − 1)] is the history of *z*_*i*_ till time *t*, and *β*_*i*,*j*_ = [*β*_*i*,*j*_(1), …, *β*_*i*,*j*_(*L*)] is the vector of coefficients modeling the effect of time series *z*_*i*_ on the target time series.

Based on this information, the time series *z*_*j*_ Granger-causes *z*_*i*_ if at least one value in the coefficient vector *β*_*j*_ is statistically nonzero.

In order to show the convergence of Copula Granger is consistent like LASSO Granger, Bahadori and Liu [24] use the mathematical concept discussed in [23, 25] which uses the linear model:

$$y=\beta^{T}x+\varepsilon$$

where **x** is a *p x* 1 is a random vector having zero mean and unit variance, *β* is the coefficient vector and *ε* is noise having zero mean and unit variance.

The real *n* observation samples ${\tilde{x}}_{i}$ f or *i* = 1,…,*n*, yields the covariance that follows the following rate as suggested by [23]

$$\underset{j,k}{\max}\left|\tilde{S_{jk}^{n}}-\hat{S_{jk}^{n}}\right|=O_{p}\left(\sqrt{\frac{\log\;p\;\log\;{}^{2}p}{n^{\frac{1}{2}}}}\right)$$

Where $\hat{S_{jk}^{n}}={\left(X^{T}\;X\right)}_{jk}$ and $\tilde{S_{jk}^{n}}={\left({\tilde{X}}^{T}\;\tilde{X}\right)}_{jk}$ is our estimate of covariance using the actual and noisy samples *x*_*i*_ and ${\tilde{x}}_{i}$ and assuming that the matrix $\Delta =\tilde{C}-C$ is positive semi-definite. This assumption is bound by following equation which is modified version of equation (22) of [25]:

$$\gamma^{T}\tilde{C}\gamma \leq \lambda \sqrt{s}\;{\left\|\gamma \right\|}_{2}+\varphi_{max}(\Delta )$$

Provided $\varphi_{max}(\Delta )\leq K_{2}max\;|\tilde{S_{jk}^{n}}-\hat{S_{jk}^{n}}|$ is bounded for some constant *K*_2_ and deriving the lower bound in Eq 26 using the fact that *φ*_*min*_(Δ) ≥ 0 yields the following equation:

$$K\emptyset_{min}{\left\|\gamma \right\|}_{2}^{2}\leq \frac{\frac{\lambda }{n}\sqrt{s}}{K\emptyset_{min}+\varphi_{max}}$$

Since *φ*_*max*_(Δ) *d*iminishes with respect to $\emptyset_{min}\;(\tilde{C})$ according to results from [23] and having the incoherent design assumption [25] for lower bound of $\emptyset_{min}\;(\tilde{C})$, the proof is established following the steps in [25].

Further mathematical details and proof of the concepts are explicitly not discussed here and can be referred to in the original article [24]. However, the gist of their technique is to isolate the marginal properties of the data from their dependency structures. In order to implement this idea, they used ℓ1 (LASSO) regularization to estimate dependency structures for high-dimensional data. In this way, they used the advantage of LASSO scalability for higher dimensions and at the same time used copulas to handle the nonlinearity of the data.

### 2.1 Limitations of Existing Work

In the standard LASSO estimator [26], the ℓ1 penalty is used to obtain the sparse solution to the following optimization problem:

$$\beta (LASSO)=\underset{\beta }{\min}{\left\|y-X\beta \right\|}_{2}^{2}+\lambda {\left\|\beta \right\|}_{1}$$

where ${\left\|\beta \right\|}_{1}=\sum_{i=1}^{p}\left|\beta_{i}\right|$ is the ℓ1-norm penalty on *β* and induces sparsity in the solution, and *λ* ≥ 0 is a tuning parameter.

The use of the ℓ1-norm penalty helps in the simultaneous operations of regularization and shrinkage and thus makes LASSO an appealing variable selection method. However, despite these advantages, LASSO faces some limitations, as discussed by Zou [27], that makes it unstable when used for high-dimensional data and limits the variable selection before saturation when the number of variables is greater than the number of observation points.

These LASSO problems mainly arise when dealing with either highly correlated predictors, which usually results in the random selection of predictors, or when all predictors are identical, as discussed in detail in Friedman, Hastie, and Tibshirani [28].

## 3 Elastic-Net Copula Granger Causality

The instabilities of LASSO can be circumvented using an extension of LASSO called elastic net. It is robust to high correlations among predictors [27] and can select more than *p* variables when *n* >> *p* (that is, the number of variables >> the number of observation points). It uses a mixture of the ℓ1 (LASSO) and ℓ2 (ridge regression) penalties and can be formulated as:

$$\beta (enet)=\underset{\beta }{\min}{\left\|y-X\beta \right\|}_{2}^{2}+\lambda_{1}{\left\|\beta \right\|}_{1}+\lambda_{2}{\left\|\beta \right\|}_{2}^{2}$$

where ${\left\|\beta \right\|}_{1}=\sum_{i=1}^{p}\left|\beta_{i}\right|$, ${\left\|\beta \right\|}_{2}^{2}=\sum_{i=1}^{p}\beta_{i}^{2}$, *λ*_1_ is the tuning parameter for ℓ1 (LASSO), and *λ*_2_ is the tuning parameter for ℓ2 (ridge regression).

As we know, ℓ2 (ridge regression) [29] works well with a large number of predictors that either have nonzero coefficients, or are drawn from a normal distribution and are highly correlated [28]. Therefore, its presence in elastic net helps improve variable selection, whereas the ℓ1 (LASSO) penalty induces the grouping effect and stabilizes the solution paths with respect to random sampling [30]. Thus, using the combination of both penalties should greatly improve the predictions.

Based on these findings, we are proposing a method called elastic-net copula Granger causality (ECGC) that we expect to be more precise than the existing GNPN method. In this new method, we employ elastic-net regularization instead of ℓ1 (LASSO) regularization to estimate dependency structures. The use of elastic net will overcome the shortcomings of the existing LASSO methods and will also exploit the advantages of copula to handle the nonlinearity of data.

Therefore, instead of solving the optimization problem proposed by Bahadori and Liu [24],i.e.,

$$\underset{\beta }{\min}\sum_{t=L+1}^{T}{\left\|X_{i}(t)-\sum_{i=1}^{p}\beta_{i,j}^{T}X_{i}^{t,Lagged}\right\|}_{2}^{2}+\lambda \;{\left\|\beta \right\|}_{1}$$

where *λ* is the tuning parameter, we are proposing to use the following optimization problem:

$$\underset{\beta }{\min}\sum_{t=L+1}^{T}{\left\|X_{i}(t)-\sum_{i=1}^{p}\beta_{i,j}^{T}X_{i}^{t,Lagged}\right\|}_{2}^{2}+\lambda_{1}\;{\left\|\beta \right\|}_{1}+\lambda_{2}{\left\|\beta \right\|}_{2}^{2}$$

where *λ*_1_ and *λ*_2_ are tuning parameters for ℓ1 (LASSO) and ℓ2 (ridge regression) penalties, respectively and are calculated using as follow: *λ*_1_ = α and *λ*_2_ = (1-α)/2. The pseudocode for implementing elastic-net copula Granger causality is summarized below:

1 Find the marginal distribution for each time series:
  $${\hat{F}}_{n}(t)=\frac{number\;of\;elements\;in\;the\;sample\leq t}{n}.$$
2 Map the observations into copula space:
  $${\hat{f}}_{i}(X_{i}^{t})={\hat{\mu }}_{i}+{\hat{\sigma }}_{i}\Phi^{-1}({\hat{F}}_{i}(X_{i}^{t}))$$

In practice, as proposed by Bahadori and Liu [17], to avoid large numbers Φ^−1^(0^+^) and Φ^−1^(1^−^) we use the Winsorized estimator of the distribution function:

$${\tilde{F}}_{j}=\left\{ \begin{array}{l} \;\delta_{n},\;if\;{\hat{F}}_{j}(X_{j})<\delta_{n}\\ \;{\tilde{F}}_{j}(X_{j}),\;if\;\delta_{n}\leq {\hat{F}}_{j}(X_{j})\leq 1-\delta_{n}\\ (1-\delta_{n}),\;if\;{\hat{F}}_{j}(X_{j})>1-\delta_{n} \end{array}\right.$$

3 Find Granger causality using elastic net and copula for different values of tuning parameters among ${\hat{F}}_{i}(X_{i}^{t})$.
4 Select Granger causality based on the minimum Akaike information criteria [31].

To evaluate our proposed method, we performed extensive experiments, which are discussed in the next section.

## 4 Experimentation and Performance Evaluation

### 4.1 Experimentation

For the implementation of our proposed method, we used MATLAB together with the Sparse Learning with Efficient Projections (SLEP) toolbox [32]. SLEP is a well-known toolbox that has functions related to regularization and has been used in the past by many researchers [33, 34].

For the comparison exercise, we used the code provided in [35], which implements LASSO copula Granger causality, and the Glmnet toolbox [36] in MATLAB.

In order to remain unbiased in our comparison, instead of using self-created data sets, we used the same data that have already been used by other authors to test similar kinds of algorithms.

#### 4.1.1 Simulated Data

The first set of data was used in [37] and [38]; it simulates the scenario of three variables (genes) and uses the following set of mathematical equations:

$$x_{1}(t)=0.8\;x_{1}(t-1)-0.5\;x_{1}(t-2)+0.4\;x_{3}(t-1)+\varepsilon_{1}\;(t)$$

$$x_{2}(t)=0.9\;x_{2}(t-1)-0.8\;x_{3}(t-2)+\varepsilon_{3}(t)$$

$$x_{3}(t)=0.5\;x_{3}(t-1)-0.2\;x_{3}(t-2)+0.5\;x_{2}(t-1)+\varepsilon_{3}(t)$$

The second set of simulated data was first used by Schelter et al. [39] and later in [18, 40, 41] and [42]. It simulates the scenario of five variables and uses the following set of equations:

$$x_{1}(t)=0.6\;x_{1}(t-1)+0.65\;x_{2}(t-2)+\varepsilon_{1}(t)$$

$$x_{2}(t)=0.5\;x_{2}(t-1)-0.3\;x_{2}(t-2)-0.3x_{3}(t-4)+0.6\;x_{4}(t-1)+\varepsilon_{2}(t)$$

$$x_{3}(t)=0.8\;x_{3}(t-1)-0.7\;x_{3}(t-2)-0.1\;x_{5}(t-3)+\varepsilon_{3}(t)$$

$$x_{4}(t)=0.5\;x_{4}(t-1)+0.9\;x_{3}(t-2)+0.4\;x_{5}(t-2)+\varepsilon_{4}(t)$$

$$x_{5}(t)=0.7\;x_{5}(t-1)-0.5\;x_{5}(t-2)-0.2\;x_{3}(t-1)+\varepsilon_{5}(t)$$

We generated the desired quantities of data from these sets of equations, as is done by other authors [18, 37, 38, 40, 41] and [42], by first initializing the equations with white Gaussian noise having zero mean and unit variance; we then used the iterative process to generate the remainder of the data.

**Fig 2:**
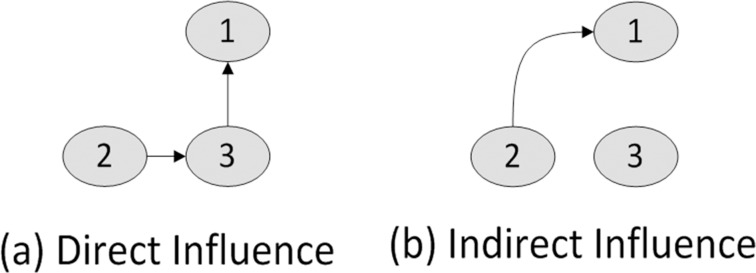
Influence graph for simulated data set 1.

**Fig 3:**
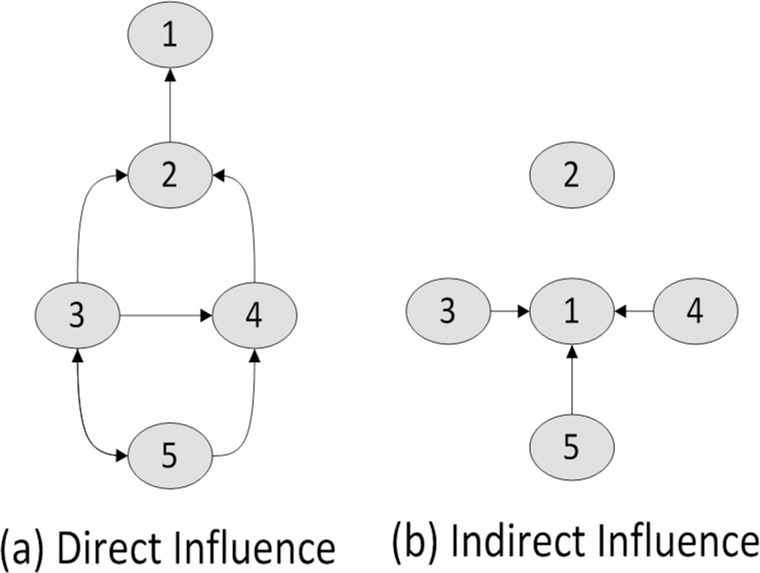
Influence graph for simulated data set 2.

The direct and indirect influence structure of the simulated autoregressive models 1 and 2 are depicted by the directed graphs in Fig 2 and Fig 3, respectively. In both figures, a directed edge represents the Granger causality between two nodes or variables.

The third simulated dataset used in this paper is from the Dialogue for Reverse Engineering Assessments and Methods (DREAM). DREAM is one of the major platforms for genetic research, in which series of challenges are given to researchers. The aim of these challenges is to provide researchers, the means to find and implement new and existing theories in the areas of cellular network inference and quantitative model building in systems biology.

The “In Silico Network Challenge,” one of the DREAM4 challenges, has the objective of inferring gene regulation network structures via reverse engineering from the given in silico gene expression data sets using any possible means. For this paper, we used the dataset for the InSilico_Size10 sub-challenge, entitled “In Silico Network of Size 10” [43–45].

#### 4.1.2 Real Data

HeLa Cancer Cell Dataset: The real data used in this paper is from the HeLa human cancer cell line that is collected by Whitfield et al. [46] by performing multiple experiments using DNA microarray analysis of the HeLa cell line. In this paper, we are using their experiment 3 data set, which has been used by other researchers as well [6, 47].

The experiment 3 data set identified more than 1100 genes that are periodically expressed during the cancer cell cycle. From these genes, we used 19 preselected genes that are regarded as highly influential and have been investigated by other researchers [6, 47, 48]. The 19 preselected genes that we considered are: “PCNA, NPAT, E2F1, CCNE1, CDC25A, CDKN1A, BRCA1, CCNF, CCNA2, CDC20, STK15, BUB1B, CKS2, CDC25C, PLK1, CCNB1, CDC25B, TYMS, and DHFR”.

As the data was not collected at homogeneous intervals, it was interpolated by cubic smoothing splines [49] before being used as advised in [6, 47].

fMRI StarPlus Dataset: The Second real dataset used in this study is called StarPlus dataset collected by [50] that can be freely accessed from [51]. This dataset had been used in past by several researchers [8, 48, 52, 53] and contained the data that was acquired to study the brain activity involved during comprehension of the relationship between sentence and pictures.

During the study, they performed series of experiments on 13 normal subjects and divided these experiments into 40 trials. In each trial, every subject had to relate a sentence with a picture and then decide the relation between sentence and picture. These 40 trials were further divided into two equal parts. In one part, they first introduced the sentence and asked to relate it to a picture whereas for the next part, they showed picture followed by a sentence.

In either setting, both stimuli were provided only for 4-second exposure and a 4-second blank screen in between. Then after the second stimulus, the subjects were asked to answer the question then rest for 15 seconds before the start of next trial. More details about experimental settings, sentences, and pictures are explicitly not discussed here and can be referred to [50].

The images that were acquired during the experiments were pre-processed using FIASCO program [54] to reduce some artifacts (signal drift and head motions) introduced during image acquisition process. Then pre-processed images were analyzed, and 25 distinct anatomical regions of interest (ROIs) were selected for further study. These regions includes: “left dorsolateral prefrontal cortex (LDLPFC) and right dorsolateral prefrontal cortex (RDLPFC), calcarine sulcus (CALC), left frontal eye fields (LFEF), right frontal eye fields (RFEF), left inferior parietal lobule (LIPL), right inferior parietal lobule (RIPL), left intraparietal sulcus (LIPS), right intraparietal sulcus (RIPS), left inferior frontal gyrus (LIFG), left opercularis (LOPER), right opercularis (ROPER), supplementary motor areas (SMA), left and right inferior temporal lobule (LIT, RIT), left and right posterior precentral sulcus (LPPREC, RPPREC), left and right supramarginal gyrus (LSGA, RSGA), left temporal lobe (LT), right temporal lobe (RT), left and right triangularis (LTRIA, RTRIA), left superior parietal lobule (LSPL) and right superior parietal lobule (RSPL)”.

### 4.2 Performance Evaluation

The performance evaluation of both methods was done by comparing our proposed method with the existing method using the following measures: precision, false detection rate (FDR), recall, and F1 score. These measures were calculated more than 5000 times for each scenario, and then their average values were used as results, to minimize impulsive errors.

**Fig 4:**
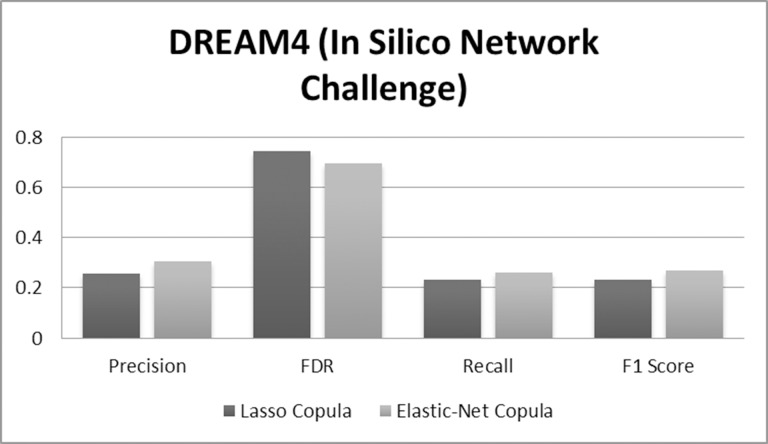
Results for DREAM4 In Silico Network Challenge.

**Fig 5:**
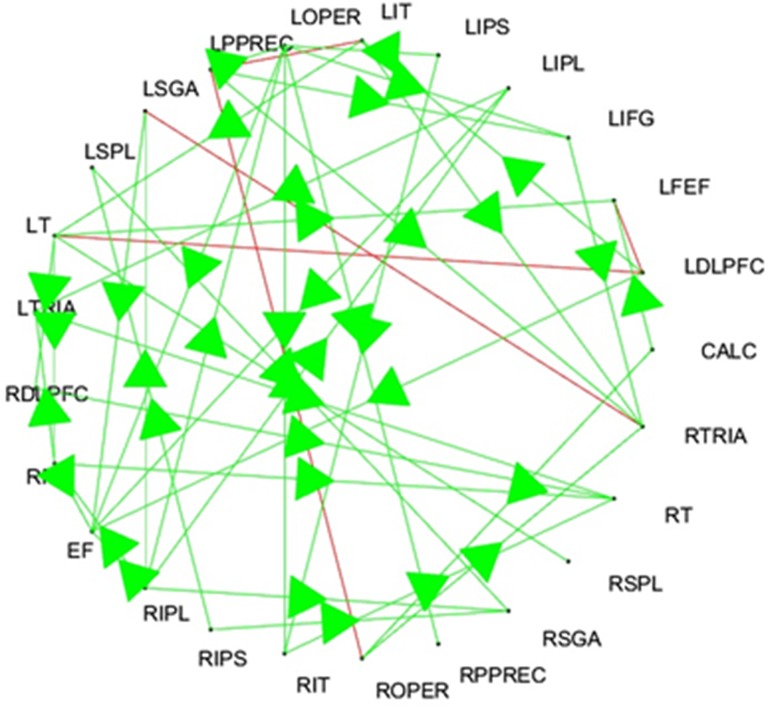
Brain Connectivity map involves in deductive reasoning.

Results for simulated data sets 1, and 2 are shown in Tables 1 and 2, respectively, whereas the results for the DREAM4 data are summarized in Fig 4. Moreover, the effective brain map involved in human deductive reasoning is shown in Fig 5.

**Table 1:** Results for Simulated Data Set 1.

*Number of Time Points*		*15*	*20*	*35*	*75*	*150*	*500*	*1000*
False Discovery Rate	*LASSO Copula GC*	0.52	0.59	0.7	0.76	0.76	0.81	0.8
	*Elastic-Net Copula GC*	0.41	0.45	0.44	0.35	0.45	0.53	0.52
Recall	*LASSO Copula GC*	0.41	0.41	0.41	0.41	0.41	0.4	0.41
	*Elastic-Net Copula GC*	0.51	0.57	0.69	0.84	0.79	0.86	0.91
F1 Score	*LASSO Copula GC*	0.42	0.39	0.33	0.3	0.3	0.26	0.27
	*Elastic-Net Copula GC*	0.51	0.53	0.58	0.70	0.64	0.61	0.62
Precision	*LASSO Copula GC*	0.48	0.51	0.30	0.24	0.24	0.19	0.20
	*Elastic-Net Copula GC*	0.59	0.56	0.56	0.65	0.55	0.48	0.48

**Table 2:** Results for Simulated Data Set 2.

*Number of Time Points*		*15*	*20*	*35*	*75*	*150*	*500*	*1000*
False Discovery Rate	*LASSO Copula GC*	0.7	0.67	0.61	0.59	0.6	0.55	0.59
	*Elastic-Net Copula GC*	0.59	0.56	0.51	0.55	0.54	0.54	0.55
Recall	*LASSO Copula GC*	0.15	0.36	0.58	0.58	0.53	0.91	0.80
	*Elastic-Net Copula GC*	0.35	0.44	0.67	0.78	0.8	0.91	0.95
F1 Score	*LASSO Copula GC*	0.19	0.33	0.46	0.48	0.46	0.6	0.55
	*Elastic-Net Copula GC*	0.36	0.42	0.54	0.57	0.58	0.61	0.61
Precision	*LASSO Copula GC*	0.31	0.33	0.39	0.41	0.4	0.45	0.42
	*Elastic-Net Copula GC*	0.41	0.44	0.49	0.45	0.46	0.46	0.45

For the real HeLa cell data, as there are no means to compare the effectiveness against other methods, we extracted the top 20 significant interactions. We looked up these 20 interactions in the BioGRID database [55] to see whether they had been reported earlier. Once all 20 interactions were analyzed, we compared the number of matches found by each method. This approach for evaluation has been used and suggested by other researchers [56]. The results of our proposed method and the LASSO copula method are summarized in Table 3.

**Table 3:** Top 20 Significant Gene Interactions using Elastic-Net Copula Granger Causality and LASSO Copula Granger Causality.

*Elastic-Net*			*LASSO*		
*Copula Granger*			*Copula Granger*		
CCNB1	↔	CDC25B	CCNB1	↔	CDC25B
E2F1	↔	CCNE1	E2F1	↔	CCNE1
CCNE1	↔	CDC25A	CCNE1	↔	CDC25A
PLK1	↔	CCNB1	PLK1	↔	CCNB1
CDKN1A	↔	BRCA1	PCNA	↔	NPAT
PCNA	↔	NPAT	PCNA	↔	PCNA
CDC25A	↔	CDKN1A	CDC25A	↔	CDKN1A
PCNA	↔	PCNA	CDKN1A	↔	BRCA1
CCNB1	↔	CCNF	BRCA1	↔	CDC25B
CDC25C	↔	PLK1	CDC25C	↔	PLK1
CDC25B	↔	TYMS	CDC25B	↔	TYMS
CCNB1	↔	STK15	NPAT	↔	E2F1
BUB1B	↔	CKS2	BUB1B	↔	CKS2
NPAT	↔	E2F1	DHFR	↔	DHFR
DHFR	↔	DHFR	CDC20	↔	CDC25B
STK15	↔	BUB1B	CCNA2	↔	CDC20
CCNA2	↔	CDC20	STK15	↔	BUB1B
CKS2	↔	CDC25C	CKS2	↔	CDC25C
PCNA	↔	E2F1	NPAT	↔	NPAT
CCNB1	↔	CKS2	BUB1B	↔	CDC25B

## 5 Discussion and Conclusion

For the evaluation of performance, we divided the simulated data into two groups: high-dimensional data and low-dimensional data. High-dimensional cases were defined as those having less than 100-time points, whereas low-dimensional cases are those having more than 100-time points. These definitions were determined based on the fact that techniques similar to DNA microarray analysis or fMRI, which can analyze multiple genes simultaneously, usually generate 15–75-time points because of their data procurement procedures.

Based on this division, the results for simulated data sets 1 and 2 show that for high-dimensional data, on the average, elastic-net copula has 14.8% better precision, 15.92% lower FDR, 19.18% higher recall, and a 16.33% higher F1 score. For the low-dimensional cases, the elastic-net copula has 16.5% better precision, 16% lower FDR, 29.3% higher recall, and a 20.75% higher F1 score.

Based on these findings on simulated data, we observe that our method is consistent with both low- and high-dimensional data with respect to precision, FDR, and F1 score. For recall, we believe that the drastic increase of performance for low-dimensional cases is due to the greater availability of information as more time point values are considered.

Similar trends for precision, FDR, recall, and F1 score can be seen from the DREAM4 data (Fig 4), where we observe improved results with the use of elastic-net copula Granger causality.

For brain connectivity map, there is no standard way to verify the resultant network other than performing some clinical trials. However, clinical trials and their results are out of scope of this paper.

However, for the results on real HeLa data (among those top 20 significant interactions listed in Table 3), we were able to detect 7 reported interactions using the elastic-net method, whereas only 5 interactions were found using the LASSO-based method. These matched interactions are shown in bold in Table 3. Of those seven interactions found by the elastic-net method, we note two interesting interactions (highlighted in Table 3) that were not detected at all by the LASSO copula method. These interactions are related to different cancer cell cycles, as reported in [57–59].

In this paper, we have proposed a new method called elastic-net copula Granger causality, which can use high-dimensional data for assessing both linear and nonlinear gene and brain networks. We have compared the performance of the new method with its predecessor, LASSO copula Granger causality. Based on the evidence from extensive experimentation, it is clear that elastic-net copula outperforms the existing LASSO copula. Moreover, when applied to real cancer cell data, it shows the capacity to detect some significant interactions that the other method is not able to detect, further reinforcing the effectiveness of our approach. Therefore, in our view, our proposed method provides a more stable regularization based technique to study gene and brain networks thus helping the researcher to manage and treat disease more meritoriously and proficiently.

## Consent and Sources of Real Data

Although this study involves human participants, formal consents or ethical committee approval is not required as experimental data used in this research is not collected by current authors and is freely accessible. HeLa cell Genetic is acquired from published article of Michael et al. and can be accessed from http://genome-www.stanford.edu/Human-CellCycle/Hela/data.shtml. Similarly, StarPlus fMRI data is acquired from published work of Mitchell et al. and can be accessed freely from http://www.cs.cmu.edu/afs/cs.cmu.edu/project/theo-81/www/.
